# Machining Safety in Dry Rough Milling of Casting Magnesium Alloy AZ91D Using Carbide End Mills with Different Geometries

**DOI:** 10.3390/ma18051104

**Published:** 2025-02-28

**Authors:** Ireneusz Zagórski

**Affiliations:** Department of Production Engineering, Mechanical Engineering Faculty, Lublin University of Technology, Nadbystrzycka 36, 20-618 Lublin, Poland; i.zagorski@pollub.pl

**Keywords:** dry rough end milling, magnesium alloys, helix angle, safety machining, time to ignition, ignition temperature, chip fractions

## Abstract

The machining of magnesium alloys poses the hazard of uncontrolled sparking or ignition. Machining safety indicators such as ignition time and temperature, unit mass of chips and their morphology are presented in this study. Different carbide end mill helix angles λ_s_ = 20° and λ_s_ = 50° were used. It was shown that the AZ91D alloy could be dry-milled within a wide range of machining parameters without the chip ignition hazard. Nonetheless, for a tool with 50° helix angle a powder chip fraction was observed. As a result of varying v_c_ and f_z_, smaller fragmentation of chips was observed for a tool with λ_s_ 20°. The most desirable leading fraction A was, respectively, up to 84% for λ_s_ 20° and up to 67% for λ_s_ 50° (in the case of v_c_ analysis); up to 87% for λ_s_ 20° and up to 83% for λ_s_ 50° (in the case of f_z_ analysis). Morphological images of chips proved the risk-free range of the tested milling conditions up to 1200 m/min and 0.03 mm/tooth (the chips were free from partial melting and burn marks). The use of a test stand demonstrated that time to ignition was an effective parameter for performing simplified simulations of chip ignition.

## 1. Introduction

Machining processes for machine and device components can be divided depending on the type of machining into rough machining (e.g., HPC processes) [1], finishing machining (HSC processes), precision machining, micromachining, and—which is a recent development—nanomachining. In terms of safety, the cutting zone temperature is considered to be the most important machinability indicator when machining magnesium alloys [2]. Many previous studies focused only on indicators such as chip temperature and tool/workpiece interface temperature [3,4,5]. An interesting research problem concerns determining the ignition point of magnesium alloys [6,7,8,9] and relating their ignition susceptibility to the formation of chips and time to their ignition [10].

A sudden increase in the temperature in the cutting zone can lead to uncontrolled chip ignition and, as a result, to fire and possible destruction of the machine tool, health hazard and material consequences, as well as legal risks [11]. These risks are particularly imminent in machining processes during which small fractions of chips are generated, particularly the so-called magnesium dust or chip powder, which poses health risk to machine operators and may cause ignition and potential damage to kinematic nodes of the machines. Therefore, it is vital that research be conducted on chip fragmentation and developing methods for reducing the potential risk of ignition during machining. Consequently, the development of a multi-stage procedure for ignition risk assessment seems to be a good and effective approach to ensuring safe operation of machine tools [12]. Table 1 summarizes selected tests (concerning machining safety issues) that were conducted on the AZ91D alloy using tools with different cutting edge geometries.

Assuming that the term ‘maintenance’ describes a series of events, phenomena occurring in a technical object (e.g., machine tool), it is possible to define safe areas, ensuring reliable and failure-free maintenance of the machine (keeping it in motion for as long as possible). As a rule, modern machine tools are devices with a significant degree of complexity in their subassemblies or components. Therefore, for the description of complex cases, the reliability of a technical object (machine) should be presented in a serial system [15]. The safety of machining and hence machine maintenance are inextricably associated with the problem of damage to individual parts and components of the machine due to, for example, machining hazard. Therefore, one measure of safety may be the risk itself (e.g., ignition, damage of a given element) and the thereto assigned probability of a given failure (losses in a time interval). In order to assess and diagnose machining safety, one can use certain measures of failure hazard and intensity of potential damage to individual components and the entire machine tool. Among the main groups of factors affecting machine operation reliability, we can distinguish the following: technological factors (e.g., heat in the cutting process, forces and vibrations), design factors (e.g., stiffness of the tool system, imbalance of rotating systems) and factors related to the culture of machine operation (e.g., monitoring the condition of the machine, using the right consumables) [12].

The problem of multi-criteria optimization of machining processes via the use of different machinability indicators has been the subject of many interesting and extensive studies. Special focus has been put on the following issues: machined surface quality [16] and surface layer examination [17,18], components of the total cutting force and wear of the cutting tool blades [19], vibrations in the cutting process, temperature in the cutting zone [20,21], shape and size of chips [10]. The indicators which are particularly important for machine tool operation safety in machining magnesium alloys are chip temperature (during machining processes) as well as the shape, size, and type of a generated chip fraction. Previous studies on the processes of turning, milling, or drilling of magnesium alloys often investigated the impact of heat generated in the cutting area adjacent to the cutting edges of the tool [22]. Thermal measurements usually included measuring the chip surface temperature in the cutting area and the average temperature on the tool flank [23,24], as well as temperature at the tool/workpiece interface [3,25]. Among various methods for determining the cutting zone temperature, the most widely used solutions include the use of infrared thermography (by optical pyrometry and thermal imaging cameras) [26,27], internal or external thermocouple (built into the tool or workpiece) [28], thermocolor methods [29], semiconductor thermometer methods, methods of creating boundaries, and methods of creating a thermal impact zone and changing the structure [22]. Moreover, the investigation of the ignition itself during machining with small machining allowances [30] is equally important. Another interesting modern and innovative approach is ignition point estimation, which is performed individually for a given type of magnesium alloy [31]. To give an example, a study [32] described the so-called criteria of ignition and ignition characteristics of magnesium alloys. Chip ignition during machining is often associated with an attempt to estimate and determine undeformed chip thickness. A decrease in the undeformed chip thickness reduces the shear angle. This can cause an increase in the shear plane temperature [23]. In turn, the form and type of chips formed as well as the type of ignition can depend, among others, on the alloy grade (and thus the chemical composition of this alloy and its additives) and technological parameters of the machining process. The potential ignition of fine chip fractions usually becomes less likely with increasing the content of alloy additives (including, Al, Cu, Zn, Ce, and Y) [8,9,33].

The risk of ignition in machining processes for magnesium alloys may occur due to, for example, a sudden increase in the cutting zone temperature [31]. The ignition point for magnesium and its alloys is usually in the range of approx. 480–650 °C. After exceeding this conventional ignition limit, small chips and so-called magnesium dust or powder chips are the first to ignite. When these products ignite, they burn quite rapidly with a bright, blinding, and difficult-to-extinguish flame. While performing machining processes, it is therefore necessary to strive for some control of the generated chip fractions. This control may enable obtaining specific chip fractions with higher ignition resistance due to, for example, their large unit mass [12]. Various methods can be used to examine the resulting ignition products (melting or burning products), including optical microscopic methods [10] or scanning electron microscopy (SEM) [34,35].

The novelty of the presented research is the new method of assessing the risk of ignition and safety during dry milling of the magnesium alloys. Modern indicators are used, which allow for the assessment of safety during dry machining. The literature review has demonstrated that there are no studies investigating the relationship between end mill geometry (e.g., rake and helix angle) and machining safety (e.g., the amount of generated chip fractions, the shape, size and mass of chips, chip ignition time, and temperature). The above-mentioned machinability indicators act as modern and innovative machining safety indicators. Another novelty in the presented research is the use of a ceramic heating plate as an element of the equipment of the test stand in order to test and analyze the time to ignition (so far, an element referred to as a “steel heating plate” has been used). To fill the gap, this study investigates the impact of cutting speeds v_c_ and feeds f_z_ on the safety of end milling using end mills with different helix angles. As highlighted in the Results and Discussion section, a fairly wide range of the investigated parameters of the end milling process can be used in practice. The proposed machining conditions do not increase significantly the risk of uncontrolled ignition during machining, hence they pose no higher risk of damage either to individual machine tool components or to the entire machine tool.

## 2. Materials, Methodology, Aims, and Scope of the Study

The principal objective of this research was to determine the impact of a cutting speed v_c_ and a feed per tooth f_z_ on the risk of chip ignition in a dry end milling process. This risk may occur when intermediate chip fractions and powder chips are formed. The impact of v_c_ and f_z_ on the chip mass and morphology (their shape, type), as well as on chip fractions and fragmentation was investigated. The percentage share of individual chip fractions (leading and intermediate fractions) was also determined and discussed. Moreover, the time to ignition for a selection of chip fractions was determined by simulating ignition during the machining process outside of the machine tool. The determination of this parameter was the secondary aim of this work. The terms and definitions such as chip fraction and fragmentation were used in compliance with [10,12]. These terms may be helpful and useful to describe resulting chip fractions. These fractions (both leading and intermediate) were distinguished depending on the mass as well as shape of individual chips. The original guidelines for chip shape and fraction analysis were obtained from the PN-ISO 3685:1996 and ISO 3685:1993 standards. However, it must be particularly emphasized that the two standards relate to steel turning processes. Moreover, the standards [36,37] stress that the proposed chip classification should be considered preliminary, presenting certain guidelines for each classification of the actual machining process under analysis. This is an important statement in the context of potential formation of many different types and kinds of chips. It therefore seems advisable that such classification be designed (as a continuation and extension of previous studies [10,12]) by analyzing the effects of machining conditions (v_c_ and f_z_) for carbide end mills with different helix angles. The importance of the chip fraction should be considered in the context of the quantity and size (e.g., mass) of chips that are created. A larger number of potentially unfavorable intermediate fractions may be detrimental to both the durability of the machine (also its components and equipment) and the health of operators.

Experiments involved conducting a high-speed dry rough milling process using end mills with two different helix angles (λ_s_ = 20° and λ_s_ = 50°). The end mills were 16 mm in diameter and had 3 blades (z = 3). Carbide tools were selected for machining due to their relatively high popularity, availability, and high quality, as well as relatively low cost. The experiments were performed on a vertical machining center, AVIA VMC 800 HS (AVIA, Warsaw, Poland). Test samples were made of casting magnesium alloy AZ91D. This grade is often used in the broadly understood machinery industry (both automotive and aviation industry). The following ranges of machining parameters were applied: a cutting speed of 400–1200 m/min and a feed of 0.05–0.30 mm/tooth. Other machining parameters were maintained constant: the axial depth of cut was 6 mm, and the radial depth of cut was 14 mm. The tools with different helix angles were balanced to G2.5 at 25,000 rev/min. For every chip fraction (leading and intermediate), chip mass measurements were repeated ten times, whereas the measurement of time to ignition was repeated five times.

**Figure 1:**
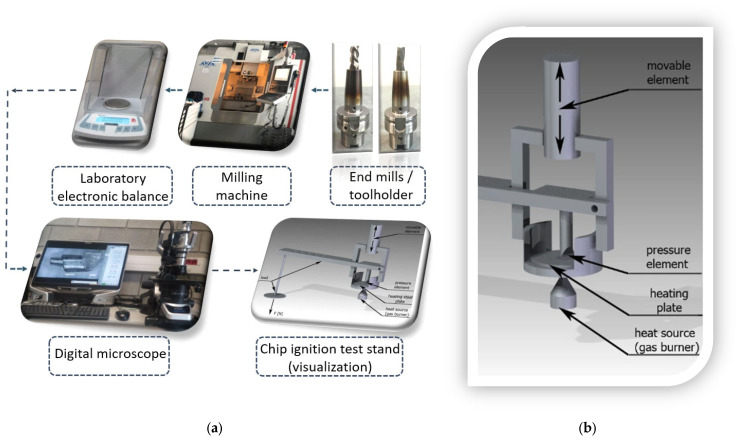
Experimental setup: (**a**) measuring equipment (e.g., milling machine, end mill with toolholder, laboratory balance, microscope for chip morphology analysis, and time-to-ignition test stand) and (**b**) chip ignition test stand with its components.

Figure 1a shows the experimental setup of measuring equipment, and the chip ignition test stand is shown in Figure 1b. The importance of the chip ignition test stand and the time to ignition should be considered in the context of potential risk and ignition hazard, and ignition simulation performed on a special test stand outside the machine tool. Such tests are all the more valuable because they do not pose a risk of damaging machine tool components or the entire machine tool due to uncontrolled chip ignition.

The following measuring equipment was used to conduct this study: a laboratory electronic balance Ohaus Discovery DV215CDM (Ohaus, Parsippany, NJ, USA) for measuring chip mass with an accuracy of 0.00001 g, digital microscope VHX-5000 KEYENCE (Keyence, Osaka, Japan) for capturing chips metallographic images, FEI NOVA NANO SEM 450 scanning electron microscope (Thermo Fisher Scientific, Waltham, MA, USA), test stand for measuring time to ignition, K type thermocouple (TP-102a-120, NiCr-NiAl) for measuring temperature, UNI-T (Dongguan, China) temperature meter (UT-320, measuring accuracy of ±[0.2% + 0.6 °C]), high-speed camera Phantom 9.1 (Adept Turnkey, Dey Road Wayne, NJ, USA) for recording chip ignition-preceding stages.

## 3. Results

This section reports the results of chip fractions and percentage share of their population, unit chips mass, time to ignition and ignition temperature, chip morphology, and ignition preceding stages.

### 3.1. Fractions and Percentage Share of Chips

Table 2 and Table 3 list obtained fractions of chips. The process was conducted using different helix angle end mills and variable cutting speeds. For most cases, the presented chips have the shape resembling short tubular, conical, or helical chips. For fraction C (helix angle 20°), the shape of chips can be defined as short and tubular. The most desirable leading fraction A was, respectively, from 8 to 84% for λ_s_ 20° and from 31 to 67% for λ_s_ 50° (in the case of v_c_ analysis). The leading chip fractions amount to approx. 80% (for a significant proportion of cases) for the 20° helix angle end mill. A rather interesting, though undesirable, exception is the situation for 400 m/min, when the A fraction is only 8%. Nevertheless, under these milling conditions, there is a fairly large amount of B fraction, with a significant unit mass (approx. 0.02 g). Moreover, with the use of the 20° helix angle tool, no chip powder fraction can be observed, which is particularly important for the proper operation of machine tools as well as the health of their operators. Unfortunately, under certain machining conditions, the use of the 50° helix angle tool leads to the creation of unfavorable chip powder.

**Figure 2:**
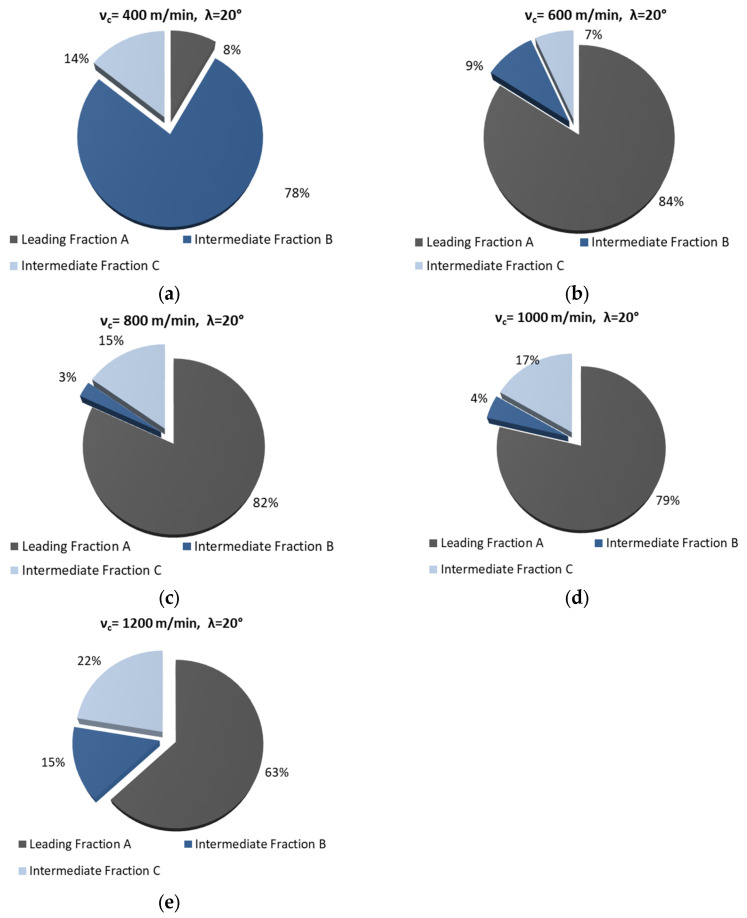
Percentage of chip fractions (leading and intermediate) obtained with different cutting speeds and a helix angle of 20°: (**a**) 400 m/min, (**b**) 600 m/min, (**c**) 800 m/min, (**d**) 1000 m/min, (**e**) 1200 m/min.

**Figure 3:**
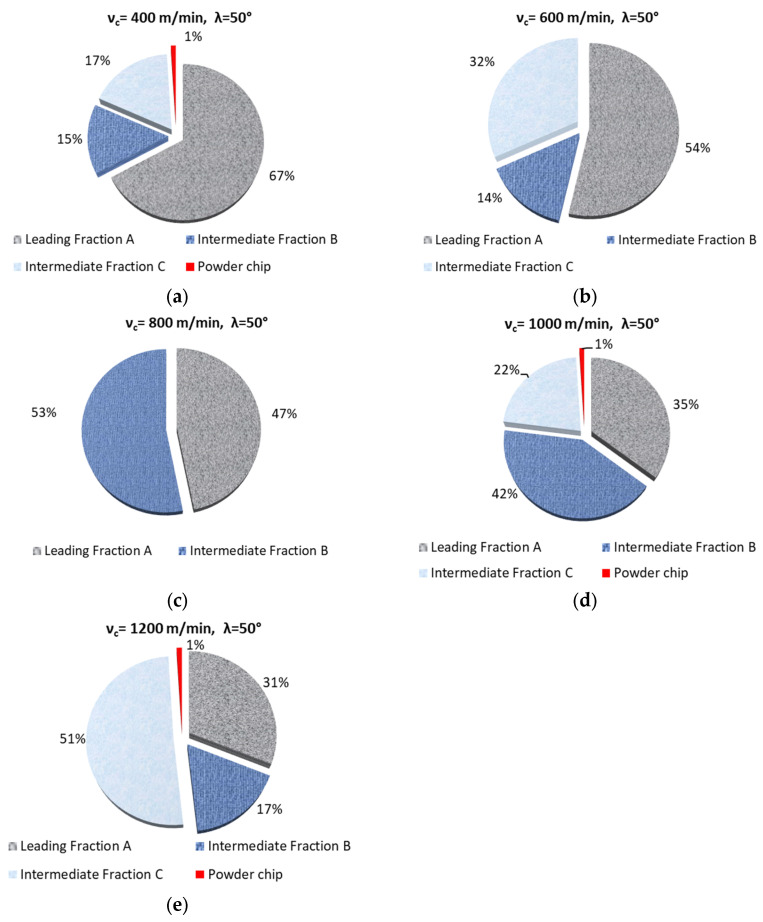
Percentage of chip fractions (leading and intermediate) obtained with different cutting speeds and a helix angle of 50°: (**a**) 400 m/min, (**b**) 600 m/min, (**c**) 800 m/min, (**d**) 1000 m/min, (**e**) 1200 m/min.

Figure 2 and Figure 3 show the percentage of chip fractions obtained with different cutting speeds. Figure 2 shows the fractions obtained with the 20° helix angle end mill, while Figure 3 shows those obtained with the 50° angle helix tool.

From a machining safety point of view, the best and most desirable solution is a milling process which produces the largest possible number of leading chip fraction. This situation occurs in milling conducted with the 20° helix angle and v_c_ ranging 600–1200 m/min. Similar cutting conditions were obtained with a helix angle of 50° and v_c_ of 600 and 800 m/min. In the remaining cases (for a helix angle of 50°), one can, however, observe the formation of powder chip fractions. In terms of machining safety, these machining conditions are undesirable.

Table 4 and Table 5 show the chip fractions obtained in a rough milling process for different feeds per tooth and helix angles of the end mills.

**Figure 4:**
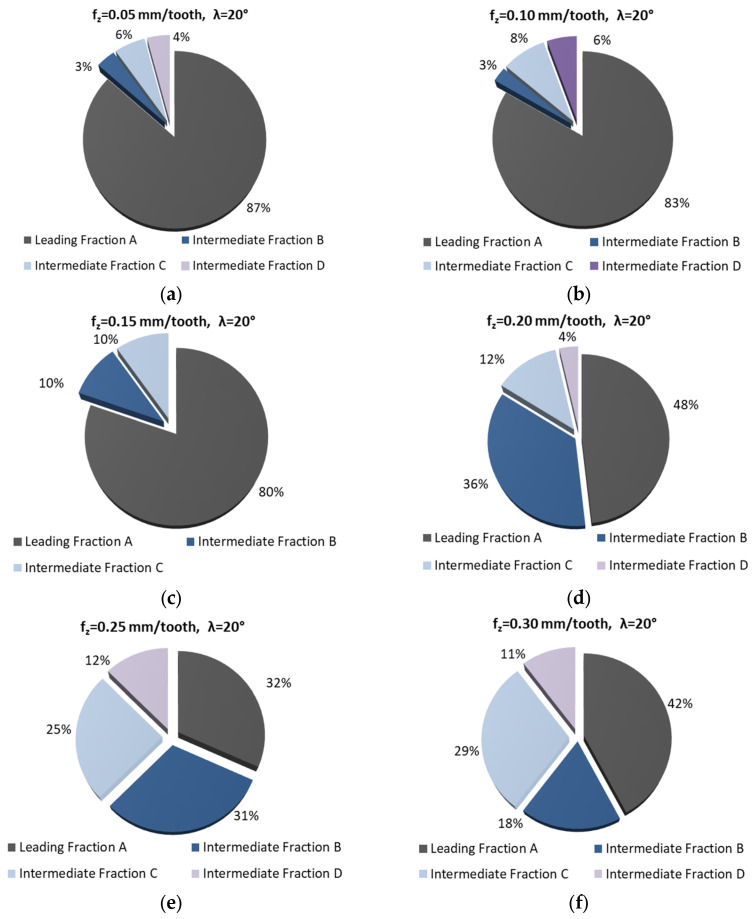
Percentage of chip fractions (leading and intermediate) obtained with different feeds per tooth (mm/tooth) and a helix angle of 20°: (**a**) 0.05, (**b**) 0.10, (**c**) 0.15, (**d**) 0.20, (**e**) 0.25, (**f**) 0.30.

**Figure 5:**
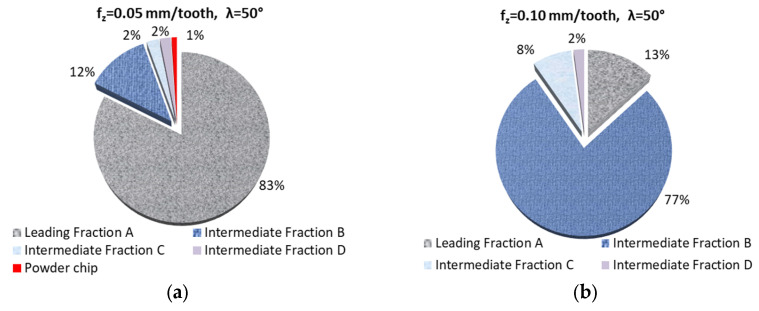

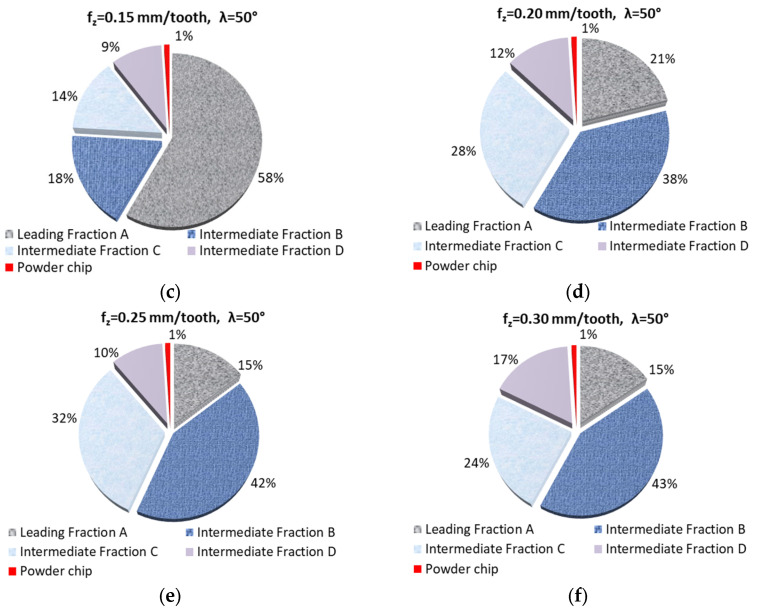
Percentage of chip fractions (leading and intermediate) obtained with different feeds per tooth (mm/tooth) and a helix angle of 50°: (**a**) 0.05, (**b**) 0.10, (**c**) 0.15, (**d**) 0.20, (**e**) 0.25, (**f**) 0.30.

Figure 4 and Figure 5 show the percentage of chip fractions obtained with different feeds per tooth. Figure 4 shows the fractions obtained using the 20° helix angle end mill, while Figure 5 shows those obtained with the 50° angle helix tool.

An analysis of the chip fractions obtained with different feeds per tooth reveals the presence of a larger number of chip fractions (especially intermediate fraction D and powder chip fractions) than was the case with variable cutting speeds. It can therefore be assumed that feed has a greater impact on the creation of a larger number of chip fractions than cutting speed.

As the feed is changed (increased), there is a greater variety in the shape of the chips. Initially (for smaller feed per tooth values), the chips are short tubular, conical, or helical. As the feed per tooth increases, the chips become less helical, with their shape resembling a loose arc. Other smaller chip fractions (e.g., fraction D) can be defined as standard needle chips.

The most desirable leading fraction A was, in this case, as follows: from 32 to 87% for λ_s_ 20° and from 13 to 83% for λ_s_ 50° (in the case of f_z_ analysis). A higher percentage of these fractions was observed for the 20° helix angle tool, especially for lower feed values (0.05–0.15 mm/tooth). In the case of the tool with helix angle 50°, fraction A was in the minority for the majority of machining cases (Figure 5b,d–f). Moreover, again, for the 20° helix angle end mill no chip powder fraction was observed. Powder chip fractions were produced when the 50° helix angle tool was used. One should also note the presence of a higher percentage of smaller chips, which are referred to as intermediate fractions.

As both v_c_ as well f_z_ are increased, the formed chips no longer have a “compact” shape; their surface has a more distorted structure (or even ragged), and less chip curl is observed.

### 3.2. Chips Mass

**Figure 6:**
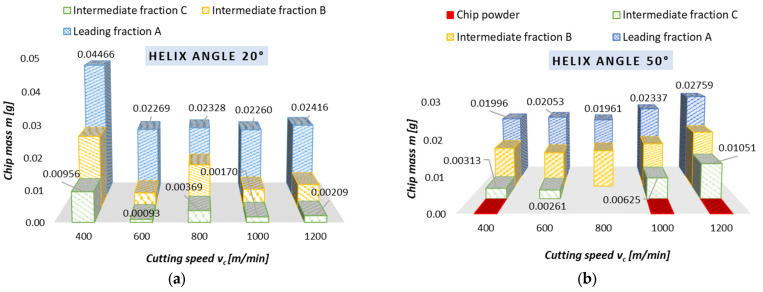
Chip mass obtained with variable cutting speed in dry rough milling conducted using (**a**) 20° helix angle and (**b**) 50° helix angle.

**Figure 7:**
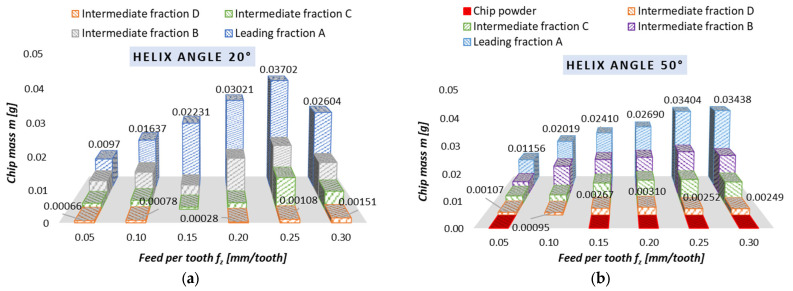
Chip mass obtained with variable feeds per tooth in dry rough milling conducted using (**a**) 20° helix angle and (**b**) 50° helix angle.

Figure 6 and Figure 7 show the chip mass results obtained for dry milling, conducted for different helix angle end mills. These results are plotted as the variables v_c_ (Figure 6) and f_z_ (Figure 7). The chip mass becomes stable as the cutting speed is changed. This can particularly be noticed for the following machining conditions: the cutting speed ranging 600–1200 m/min, helix angle of 20°, and leading chip fraction (average chip mass of approx. 0.023 g); the cutting speed ranging 400–800 m/min, helix angle of 50°, and leading chip fraction (average chip mass of approx. 0.020 g). Unfortunately, the use of the 50° helix angle tool produces a fraction known as chip powder, which is obviously an unfavorable phenomenon.

With a variation in the feed, one can observe the following dependence: an increase in the f_z_ value causes (in most cases) an increase in the percentage of the leading chip fraction, practically across the entire tested parameter range (0.05–0.25 mm/tooth). The average chip mass for the leading fraction ranges 0.0097–0.03702 g for the 20° helix angle tool and 0.01156–0.03438 g for the 50° helix angle tool. Unfortunately, as with the variable v_c_, the use of variable feeds per tooth and the 50° helix angle tool gives rise to the unfavorable phenomenon of powder chip fraction formation.

The most favorable machining conditions are those in which the smallest quantity of intermediate chip fractions is formed. This situation occurs when:

- there are 2 chip fractions (for a helix angle of 50° and v_c_ of 800 m/min),
- there are 3 chip fractions (for a helix angle of 20° and the entire tested range of v_c_, for f_z_ of 0.15 mm/tooth, for a helix angle of 50° and v_c_ of 600 m/min),
- there are 4 fractions of chips (for a helix angle of 50° and f_z_ of 0.10 mm/tooth).

### 3.3. Time to Ignition, Temperature of Ignition

**Figure 8:**
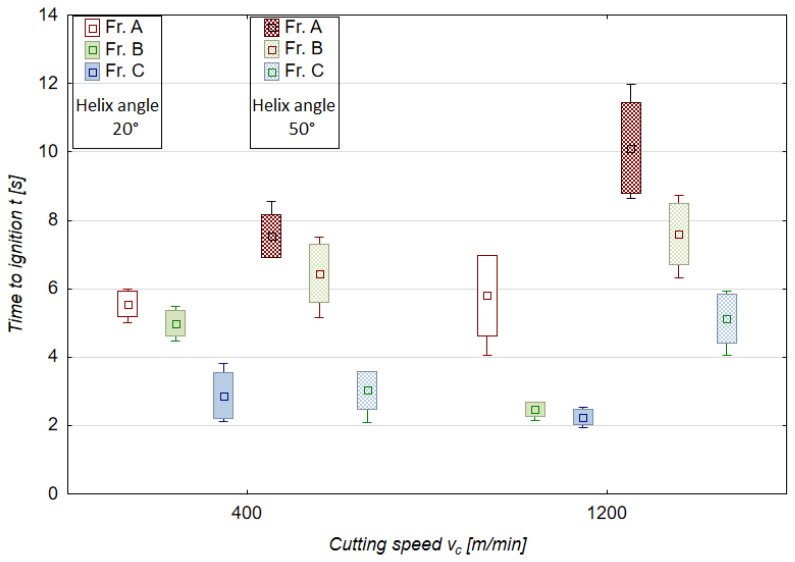
Time to ignition of magnesium alloy chips for different cutting speeds and helix angles (f_z_ 0.15 mm/tooth, a_p_ 6.0 mm, T_av_ = 507.1 °C, T_max_ = 515.1 °C).

**Figure 9:**
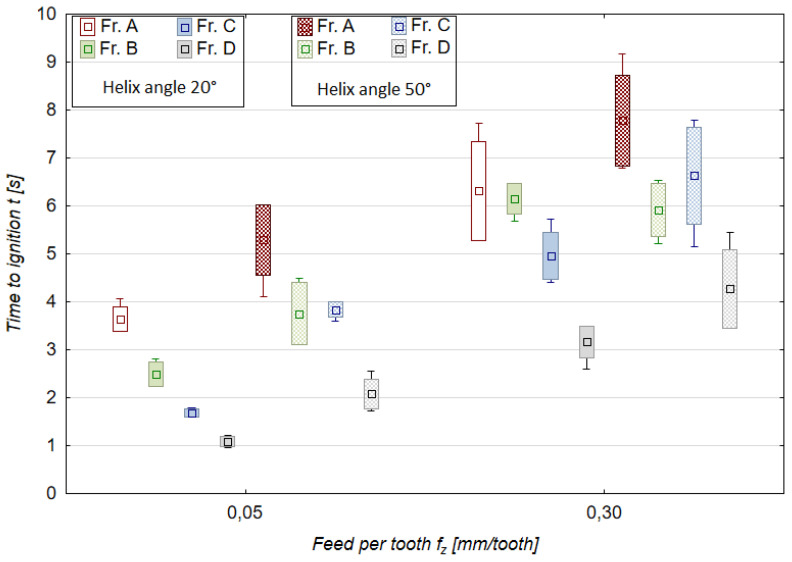
Time to ignition of magnesium alloy chips for different feeds per tooth and helix angles (v_c_ 800 m/min, a_p_ 6.0 mm, T_av_ = 509.9 °C, T_max_ = 514.9 °C).

Figure 8 and Figure 9 show the results of another important machining safety indicator, namely time to ignition. The results are presented for two extreme values (minimum and maximum) of v_c_ and f_z_. This approach was dictated by a relatively large number of different chip fractions. The time-to-ignition results are therefore plotted for all chip fractions obtained with v_c_ of 400 m/min and 1200 m/min and f_z_ of 0.05 mm/tooth and 0.30 mm/tooth.

The time-to-ignition values are higher for the A-fraction chips (leading). This is probably related to the greater mass of the A-fraction chips and the longer time that is required both for chip plasticization and for reaching a later stage of ignition. As already mentioned in [10], the time taken to form the chips is reasonably quick compared to the time to ignition observed in the experiment conducted on the special test stand outside of the milling machine. The chip formation time for the tested range of cutting speeds (up to 1200 m/min) varies between 0.01 and 0.001 s. For the results presented in Figure 8 and Figure 9, this time ranges from about 2 to 12 s for variable cutting speeds and from about 1 to 9 s for variable feeds per tooth. It is, therefore, many times longer than the chip formation time. Nevertheless, special attention should be paid to the intermediate fractions C and D because their time to ignition was the shortest, ranging from 1 to 2 s, which, combined with the small unit mass of these chip fractions, can pose a real risk of ignition during machining.

### 3.4. Chip Morphology, Chip Ignition Stages

**Figure 10:**
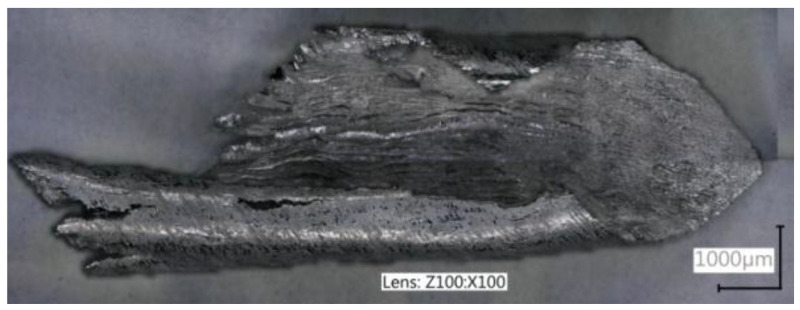
Example of a chip image captured with the VHX-5000 KEYENCE microscope.

This study also presents examples of magnesium chip images that were captured by metallographic and SEM methods. Figure 10 shows an example of a metallographic image of a chip generated during roughing, using tools with different geometries.

**Figure 11:**
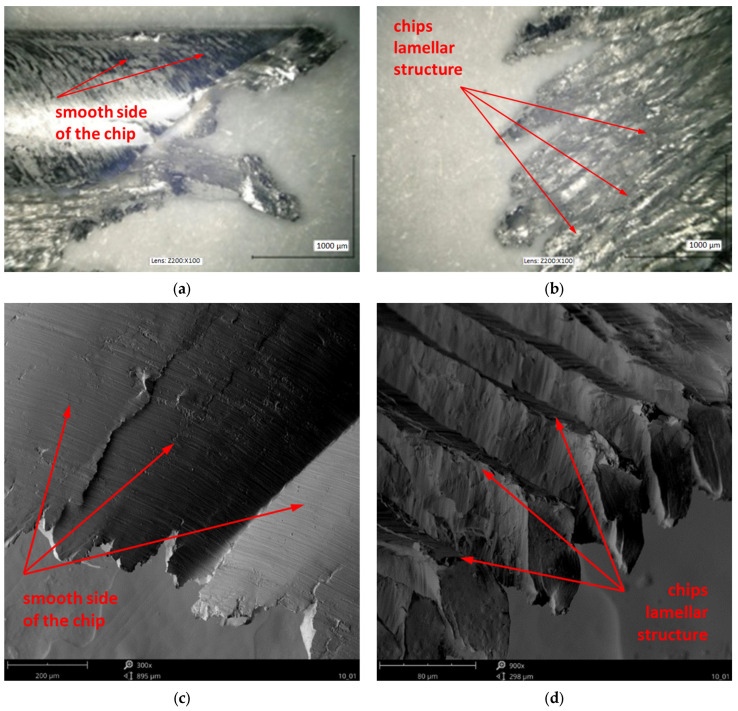
Examples of morphology and metallographic images of chip surface and their edges—a smooth surface of the chip created as a result of interaction with the surface of an end mill rake angle and a lamellar structure resulting from the impact of a subsequent shear plane: (**a**,**b**) at 100 × 100 image resolution, captured with VHX-5000 KEYENCE, (**c**,**d**), SEM image captured with FEI NOVA NANO SEM 450 (λ_s_ 50°), (**a**,**c**) ν_c_ 1200 m/min, and (**b**,**d**) f_z_ 0.3 mm/tooth).

Figure 11 shows the surface elements of the chips that were captured by metallographic and SEM techniques. The chips have a characteristic structure, consisting of so-called lamellar plate structures on one side and a smooth and shiny surface on the other side. On the magnesium alloy chip surface, one can usually distinguish elements such as lamellas (regular layered structures that are usually arranged parallel to each other, illustrating individual shear bands, Figure 11b,d) and a chip smooth side (illustrating the contact between the chip and tool rake face, Figure 11a,c). Both the edges and the surface of the chips are free from a characteristic cauliflower-like area, ignition products, or the area of intense oxidation.

**Figure 12:**
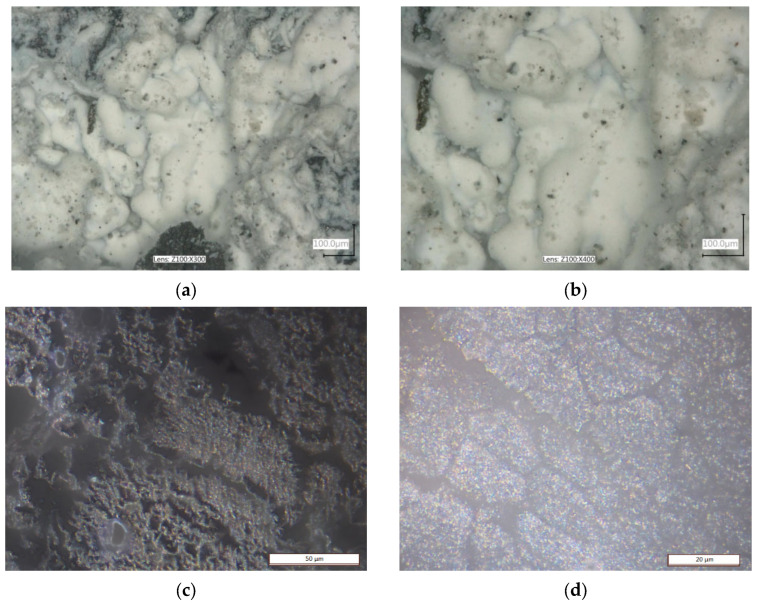
Chip surfaces: (**a**,**b**) subjected to ignition and with the presence of intense oxidation products in the form of a characteristic cauliflower-like area, (**c**,**d**) where ignition did not occur.

**Figure 13:**
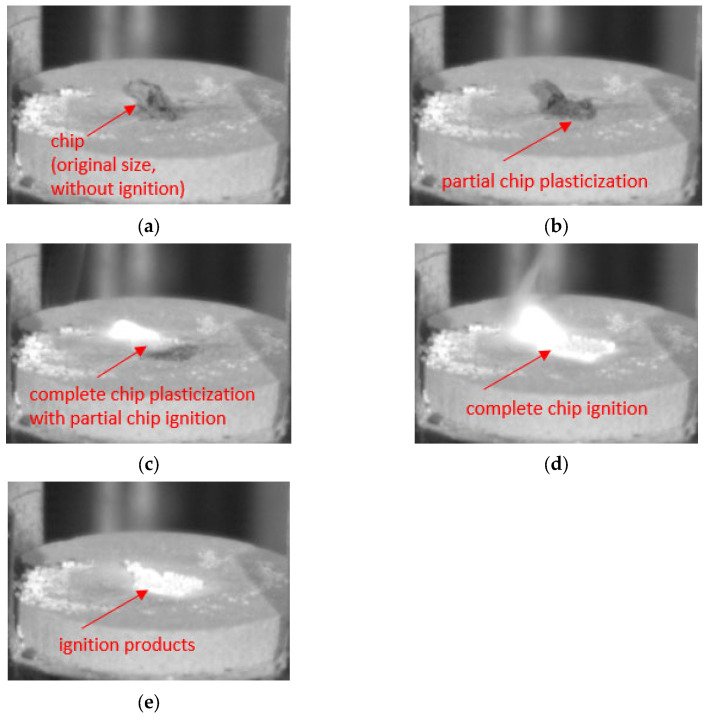
Examples of chip ignition-preceding stages identified on a test stand with a ceramic heating plate: (**a**) initial stage of chip contact with a heating ceramic plate, (**b**) incomplete plasticization, (**c**) entire plasticization with partial ignition, (**d**) entire ignition, (**e**) ignition results (with a cauliflower-like chip area), captured with a Phantom camera.

For comparison purposes, successive images show examples of chip surfaces subjected to ignition or intense oxidation. These images show the chip surface with the presence of characteristic products resembling a cauliflower area (Figure 12a,b), as well as the surface subjected to intensive oxidation without the presence of any ignition products (Figure 12c,d). In Figure 13, the stages preceding chip ignition due to the contact with the surface of a heating plate are shown. The figure shows the initial stage of chip contact, subsequent stages of plasticization and ignition, as well as the formation of burning ignition products (in the form of a cauliflower-like area).

The results and metallographic images of chips make it possible to identify areas which pose the risk of ignition. It is also possible to indicate areas in which the ignition risk is relatively low and can be prevented, e.g., by eliminating chip powder fraction.

## 4. Discussion

Previous research on ignition risk and safety in machining processes for magnesium alloys has primarily focused on the impact of rake angle. In addition, the influence of machining parameters or machining conditions on machining safety has often been analyzed. In this work, the research scope was extended to include the influence of technological dry milling parameters and end mill geometry (helix angle) on machining safety and machine tool reliability. To our knowledge, no previous studies have examined the impact of tool blade geometry on chip fraction formation and magnesium alloy machining safety. Studies have shown that the most frequently analyzed material is the casting magnesium alloy AZ91D/HP, which may result from its wide use.

Previous studies tested the outcome of different rake angles and technological milling parameters on chip formation and fragmentation [13], chip mass, as well as the chip width-to-length ratio [12]. A study [14] continued the investigation of AZ91D/HP alloy chip fractions and variable technological parameters (v_c_ and f_z_) for different rake angles (5° and 30°). The following were discussed: chip fractions, overall dimensions, and the percentage of individual chip fractions. A more recent study [10] determined the influence of a_p_ and λ_s_ on fractions of chips and their mass, temperature, and time to ignition. Moreover, the study showed selected metallographic images of chips and their edges, chips exposed to burning or heavy oxidation, and chip ignition-preceding stages.

A study [13] found that reduced fragmentation of chips was (usually) associated with an increase in a_p_. The chip fraction percentage was lower for the 30° rake angle tool. The scope of the research was then extended [12] to include an analysis of, among others, the effect of technological parameters (v_c_ and f_z_). In addition, chip fraction dimensions and their unit mass were analyzed. It was established that smaller fragmentation of chips was produced for the 30° rake angle. Furthermore, the smallest quantity of fractions was observed when varying the cutting speed. For reliability reasons, it was recommended to avoid the use of low values of both a_p_ and λ_s_. Similar conclusions were reported in [14], where smaller fragmentation and quantities of chip fractions were produced with the 30° rake angle. The results demonstrated that the highest possible values of f_z_ and a_p_ should be used, although chip fragmentation increased with increasing v_c_ and f_z_. In turn, varying the helix angle [10] affected the unit mass of chips, with higher chip mass values observed for the 50° helix angle. Favorable machining conditions occurred when a_p_ was equal to 6 mm, resulting in the presence of a large amount of the leading fraction (fraction A). Moreover, these conditions were also found to be favorable in terms of machining efficiency and productivity. The time needed for chip ignition was much longer than the time of chip formation during machining.

This study examined the problem of chip fragmentation for variables v_c_, f_z_, and different λ_s_. The study can be considered supplementary to the field of the machinability of casting magnesium alloy AZ91D. It was observed that no dust fraction occurred for a tool with a 20° helix angle. Moreover, for most cases, a smaller number of predominantly intermediate chip fractions was observed with varying v_c_ rather than f_z_, despite using different end mill geometries. The time to chip ignition parameter is an important indicator of machining safety, and—similarly to previous works [10]—the present study showed this parameter to be several times longer than the time required for chip formation in the cutting process.

## 5. Conclusions

Based on the results obtained from this research, the following conclusions can be drawn:

- It is possible to implement safe, effective, as well as reliable dry milling of magnesium alloys, and therefore ensure safe maintenance of machine tools without a potential risk of damaging their components.
- The best and most preferred machining conditions are those which generate only two or three chip fractions (e.g., full range v_c_ and f_z_ for 20° helix angle tool).
- Machining conditions where four chip fractions occur but no chip powder is present can be considered acceptable; such conditions can be observed for 50° helix angle when v_c_ equals 600 and 800 m/min (see Figure 6b), and when f_z_ equals 0.10 mm/tooth (see Figure 7b).
- The use of the 20° helix angle tool produces no chip powder, a product which is considered the most harmful and potentially dangerous chip fraction; this tool should therefore be the first choice for rough machining of magnesium alloys.
- The most advantageous machining areas are those where the A fraction constitutes about 50% of the share in all chip fractions; this situation occurs for the entire v_c_ range (without v_c_ equal to 400 m/min) for λ_s_ = 20° and v_c_ 400–800 m/min for λ_s_ = 50°; the range 0.05–0.20 mm/tooth for λ_s_ = 20° and for 0.05 and 0.15 mm/tooth for λ_s_ = 50°.
- The unit mass of chips is an important indicator for estimating machining safety in terms of ignition susceptibility of individual chip fractions; unit chip mass of fraction A is within the following ranges: for v_c_ and λ_s_ = 20—from 0.02260 to 0.04466 g; for v_c_ and λ_s_ = 50°—from 0.01961 to 0.02759 g; for f_z_ and λ_s_ = 20°—from 0.00970 to 0.03702 g; for f_z_ and λ_s_ = 50°—from 0.01156 to 0.03438 g.
- For the 50° helix angle tool, it was observed that the use of higher machining parameters led to a higher unit mass of leading fraction A (a similar trend was observed for the 20° helix angle tool and the feed of 0.25 mm/tooth).
- The longest time to chip ignition was observed for leading fraction A, and it should be emphasized that the ignition of intermediate fraction D was not immediate (the chips ignited after approx. 1–3.5 s for the variable feed per tooth and after 2–4 s for the variable cutting speed), which is an important finding regarding ignition susceptibility and sudden ignition hazard when carrying out machining operations.
- Both time to ignition as well as ignition temperature (determined outside of the milling machine, at a specially designed and constructed test stand) are important safety indicators, as they make it possible to estimate machining conditions that are considered safe working areas for a given machine tool.
- The AZ91D alloy chip surfaces (obtained by SEM and metallographic examination) were free from ignition or intense oxidation products and had clearly outlined edges; hence, under the presented machining conditions, no risk of chip ignition was observed during machining.

Future studies, which will further broaden the existing scientific knowledge and contribute to the development of the mechanical engineering discipline, should investigate the machinability of the AZ31B alloy in roughing conditions. Future research should also investigate finish milling and precision milling processes as new directions of scientific research in this area.

## Figures and Tables

**Table 1:** Selected research on the influence of tool geometry on machining safety indicators.

Machining Conditions	Research Object, Machinability Indicators	Reference
Dry Rough Down End-Milling (different helix angles): a_p_ = 0.5–6.0 mm, f_z_ = 0.15 mm/tooth, v_c_ = 800 m/min, a_e_ = 14 mm	Fractions of chips and their metallographic images, chip mass, percentage share of chip fractions, time to ignition, ignition temperature, stages preceding chip ignition	[10]
Dry Rough Down End-Milling (different rake angles): a_p_ = 0.5–3.0 mm, f_z_ = 0.05–0.30 mm/tooth, v_c_ = 400–1200 m/min, a_e_ = 14 mm	Fractions of chips and their metallographic images, chip mass, dimensions	[12]
Dry Rough Down End-Milling (different rake angles): a_p_ = 0.5–3.0 mm, f_z_ = 0.05 and 0.15 mm/tooth, v_c_ = 800 m/min, a_e_ = 14 mm	Fractions of chips and their metallographic images	[13]
Dry Rough Down End-Milling (different rake angles): a_p_ = 6.0 mm, f_z_ = 0.05–0.30 mm/tooth, v_c_ = 400–1200 m/min, a_e_ = 14 mm	Fractions of chips and their metallographic images, chip mass, percentage share of chip fractions, dimensions of chips, share of individual fractions in the total chip population	[14]

**Table 2:** Types of chip fractions obtained with different cutting speeds and a helix angle of 20°.

Type of Chip Fraction	Cutting Speed v_c_ [m/min]				
	400	600	800	1000	1200
Leading fraction A	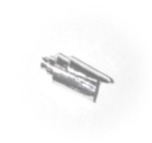	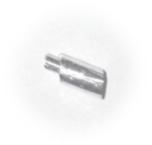	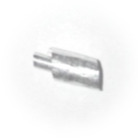	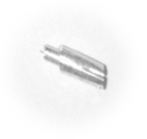	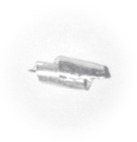
Intermediate fraction B	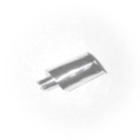	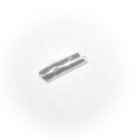	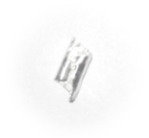	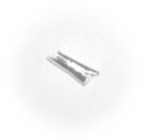	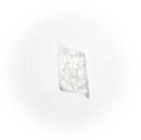
Intermediate fraction C	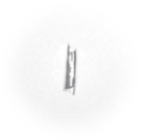	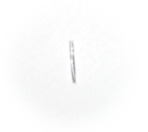	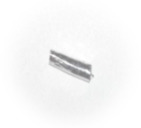	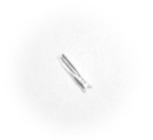	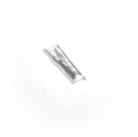
Intermediate fractions D	None	None	None	None	None
Chip powder	None	None	None	None	None

**Table 3:** Types of chip fractions obtained with different cutting speeds and a helix angle of 50°.

Type of Chip Fraction	Cutting Speed v_c_ [m/min]				
	400	600	800	1000	1200
Leading fraction A	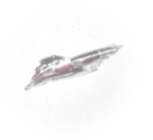	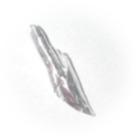	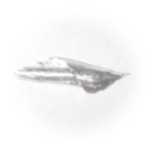	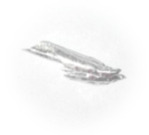	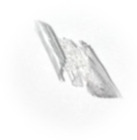
Intermediate fraction B	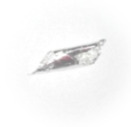	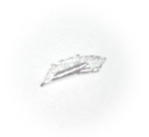	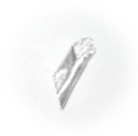	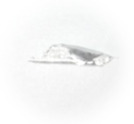	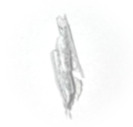
Intermediate fraction C	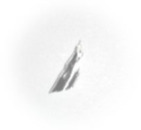	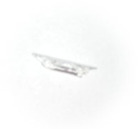	None	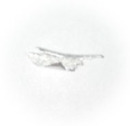	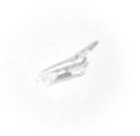
Intermediate fraction D	None	None	None	None	None
Powder chip	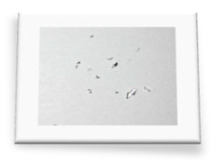	None	None	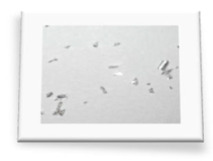	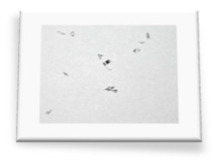

**Table 4:** Fractions of chips obtained with different feeds per tooth and a helix angle of 20°.

Type of Chip Fraction	Feed per Tooth f_z_ [mm/tooth]					
	0.05	0.10	0.15	0.20	0.25	0.30
Leading fraction A	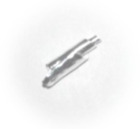	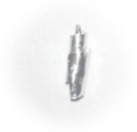	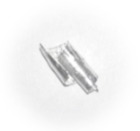	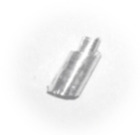	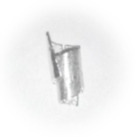	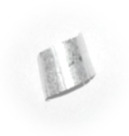
Intermediate fraction B	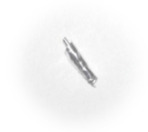	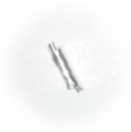	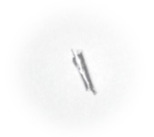	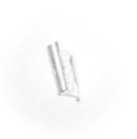	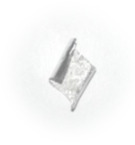	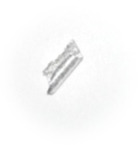
Intermediate fraction C	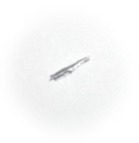	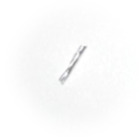	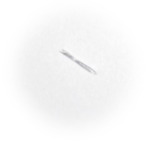	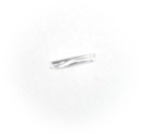	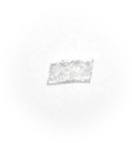	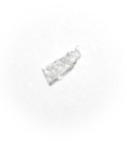
Intermediate fraction D	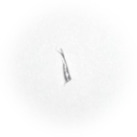	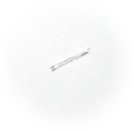	None	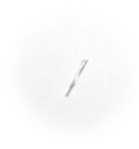	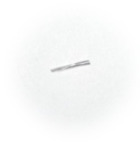	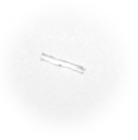
Powder chip	None	None	None	None	None	None

**Table 5:** Fractions of chips obtained with different feeds per tooth and a helix angle of 50°.

Type of Chip Fraction	Feed per Tooth f_z_ [mm/tooth]					
	0.05	0.10	0.15	0.20	0.25	0.30
Leading fraction A	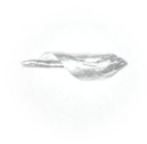	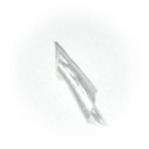	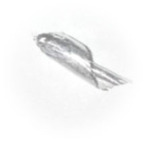	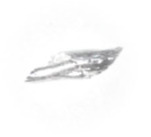	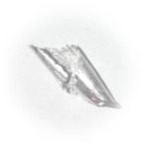	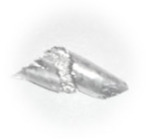
Intermediate fraction B	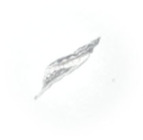	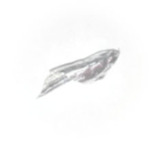	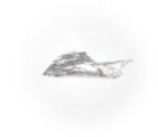	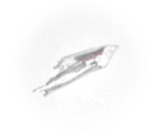	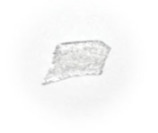	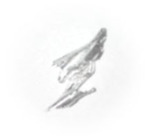
Intermediate fraction C	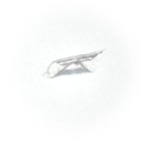	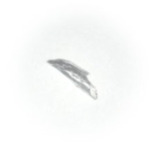	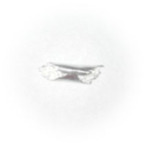	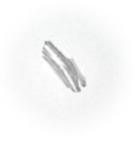	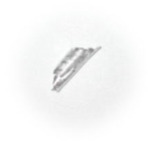	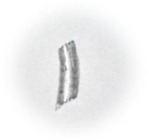
Intermediate fraction D	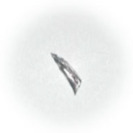	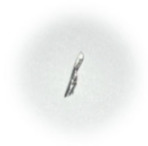	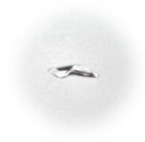	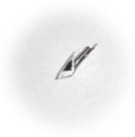	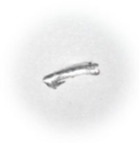	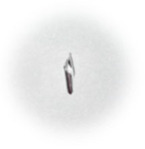
Powder chip	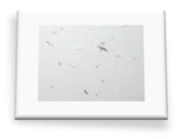	None	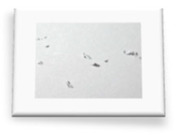	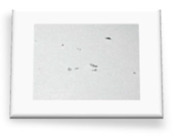	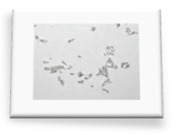	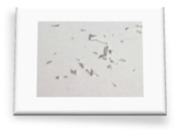

## Data Availability

The data are not publicly available due to restrictions of the study being ongoing.

## Abbreviations

The following abbreviations are used in this manuscript:

λ_s_	Helix angle
v_c_	Cutting speed
f_z_	Feed per tooth
a_p_	Axial depth of cut
a_e_	Radial depth of cut
z	Number of blade, tooth
HSM	High speed machining
HSC	High speed cutting
HPC	High performance cutting
SEM	Scanning electron microscopy
T_av_	Average temperature
T_max_	Maximum temperature
Fr. A	Chip fraction (leading A)
Fr. B	Chip fraction (intermediate B)
Fr. C	Chip fraction (intermediate C)
Fr. D	Chip fraction (intermediate D)
